# miR-520d-5p can reduce the mutations in hepatoma cancer cells and iPSCs-derivatives

**DOI:** 10.1186/s12885-019-5786-y

**Published:** 2019-06-15

**Authors:** Norimasa Miura, Yoshitaka Ishihara, Yugo Miura, Mai Kimoto, Keigo Miura

**Affiliations:** 1 PEZY-Pharma, Inc., 2-13-14 Hatagasaki, Yonago, Tottori 683-8503 Japan; 2 i-Medical Clinic, 3-4-18 Mejiro, Toshima-ku, Tokyo, 171-0031 Japan; 3 Division Pharmacotherapeutics, Faculty of Medicine, Tottori University, 86 Nishicho, Yonago, Tottori 683-8503 Japan; 4 Department of Orthopaedic Surgery, Soka Municipal Hospital, 2-21-1 Soka, Soka, Saitama 340-8560 Japan; 5 Hokkaido System Science Co., Ltd., 2-1, Shinkawa Nishi 2-1, Kitaku, Sapporo, 001-0932 Japan

**Keywords:** Mutation, iPSC, Progenitor MSC, Hsa-miR-520d-5p, Genomic conversion

## Abstract

**Background:**

Human microRNAs (miRNAs) have diverse functions in biology, and play a role in nearly every biological process. Here we report that miR-520d-5p (520d-5p) causes undifferentiated cancer cells to adopt benign or normal status in vivo in immunodeficient mice via demethylation and P53 upregulation. Further we found that 520-5p causes normal cells to elongate cellular lifetime and mesenchymal stem cell-like status with CD105 positivity. We hypothesized that ectopic 520d-5p expression reduced mutations in undifferentiated type of hepatoma (HLF) cells through synergistic modulation of methylation-related enzymatic expression.

**Methods:**

To examine whether there were any changes in mutation status in cells treated with 520d-5p, we performed next generation sequencing (NGS) in HLF cells and human iPSC-derivative cells in pre-mesenchymal stem cell status. We analyzed the data using both genome-wide and individual gene function approaches.

**Results:**

520d-5p induced a shift towards a wild type or non-malignant phenotype, which was regulated by nucleotide mutations in both HLF cells and iPSCs. Further, 520d-5p reduced mutation levels in both the whole genome and genomic fragment assemblies.

**Conclusions:**

Cancer cell genomic mutations cannot be repaired in most contexts. However, these findings suggest that applied development of 520d-5p would allow new approaches to cancer research and improve the quality of iPSCs used in regenerative medicine.

**Electronic supplementary material:**

The online version of this article (10.1186/s12885-019-5786-y) contains supplementary material, which is available to authorized users.

## Background

miRNAs are small, non-coding RNAs of only 19–25 bases in length, which control gene expression by targeting mRNA for degradation or inhibiting translation. Aberrant epigenetics and coding gene mutations are known to play a role in the development and progression of cancer [1–4]. DNA methylation, histone acetylation, histone methylation and miRNA play important roles in epigenetics [5–7]. Some miRNAs are known to be dysregulated in multiple cancers, and are classified as onco-miRs, suppressor-miRs, or metasta-miRs [8–10]. Target gene expression is negatively correlated with miRNA levels. miRNA and other epigenetic mechanisms underlie network formation, and are involved with the prevention or progression of neoplasia. Thus, examining miRNA alterations in cancers is important to predict patient outcomes and develop novel therapies. However, no previous reports have identified that miRNAs can alter differentiation in cancers. This notion is not generally accepted, but in the present report we provide supporting evidence of this phenomenon.

Next-generation sequencing (NGS) technology has produced a substantial volume of biological data, and has shed light on the path towards personalized medicine. Although the acquisition cost of high-throughput genome sequencing has decreased, the analysis and interpretation of these large-scale sequencing data continues to be prohibitive [11–13]. Many aligner and variant programs have been developed to identify variants in GS data, which have been developed and incorporated into diverse pipelines. A typical pipeline contains an aligner and a variant program, wherein the aligner program maps the sequencing reads to a reference genome, and the variant program identifies variant sites and assigns a genotype to the subject(s). The performances of different aligners have been studied extensively [11, 14, 15]. NGS is a powerful tool for identifying rare and de novo variants, disease mapping, and quantification of expression levels. For analysis, NGS reads are first aligned to a reference genome, and subsequently subjected to variant analysis after necessary quality control procedures. Whole exome sequencing of thousands of human cancers has led to the unexpected discovery of many inactivating mutations of genes that control the epigenome [16]. These mutations have the potential to disrupt DNA methylation patterns, histone modifications, and nucleosome positioning and therefore, gene expression. Genetic alteration of the epigenome therefore contributes to cancer, just as epigenetic processes can cause point mutations and impairment of DNA repair functions.

We previously reported that 520d-5p can convert undifferentiated status of cancer to benign or normal status via demethylation and P53 upregulation, hence reprogramming cancer cells to human mesenchymal stem cells (hMSCs) in conditions that can maintain differentiation [17, 18]. Furthermore, 520d-5p reprograms fibroblasts into CD105-positive cells, extending cellular lifespan [19] and preventing fatal DNA damage via a non-canonical nuclear stress and demethylation process [20]. Therefore, we postulated that this small molecule may alter epigenetic status, and in the present study evaluated 520d-5p influence on mutations in cancer cells using NGS, including exome analysis.

## Methods

### Cell preparation

For DNA extraction, we used three cell lines and lentiviral vectors. hiPSCs (HPS0002: 253G1), mesenchymal stem cells (hMSC), and undifferentiated type of hepatoma cells (HLF) were provided by the RIKEN BioResource Center Cell Bank [21] and the Cell Resource Center for Biomedical Research, Institute of Development, Takara Bio (Kusatsu, Shiga, Japan), and Aging and Cancer Tohoku University, respectively. HLF, MSC, and iPSC (253G1) were cultured in RPMI1640 medium supplemented with 10% FBS and 1% penicillin/streptomycin, in Mesenchymal Stem Cell Growth Medium 2 (Takara Bio, Kusatsu, Shiga, Japan), and in ReproStem medium (ReproCell, Tokyo, Japan) with 10 ng/ml of bFGF-2, respectively. Additionally, for the transfection to HLF cells or hMSCs, the human mesangial cell line 293FT (Invitrogen Japan K.K., Tokyo, Japan) was used for producing 520d-5p expressing-lentiviral particles. 293FT cells were cultured in DMEM supplemented with 10% FBS, 0.1 mM MEM nonessential amino acid solution, 2 mM L-glutamine and 1% penicillin/streptomycin. Also, we induced hMSCs from iPSCs using STEMdiff Mesenchymal Progenitor kit (STEMCELL technologies, Seattle, WA, USA) and defined them as 520d/MSC after they were transfected by miR-520d-5p.

### Lentiviral vector construct

To examine the effects of miR-520d-5p overexpression, we transfected pMIRNA1-miR-520d-5p/GFP (20 μg; System Biosciences, Mountain View, CA, USA) or the mock vector pCDH (20 μg) into 293FT and HLF cells (5 × 10^6^ cells/10 cm culture dish). To investigate the HLF cells and hMSCs with miR-520d-5p expression, we harvested viral particles produced in the medium cultured 293FT cells, the cells were centrifuged at 170,000 x g (120 min, 4 °C). The viral pellets were collected, and viral copy numbers were measured with a Lenti-X™ qRT-PCR Titration kit (Clontech, Mountain View, CA, USA). For 293FT or HLF cell infection, one million lentiviral copies were used per 10-cm culture dish. We transfected 50 nM synthesized oligonucleotides into 293FT cells with FuGENE HD Transfection Reagent (Roche Diagnostics, Basel, Switzerland). pCDH/lenti/GFP-treated cells were used as controls. 520d-5p-transfected hMSCs were generated by the lentiviral transfection of 520d-5p to hMSCs after the differentiation induction from iPSCs to hMSCs using hPSC-Derived MSC differentiation system and reagents (Veritas Corporation, Tokyo, Japan).

### Next-generation sequencing (NGS) analysis

The following cell lines were used in NGS analysis: HLF, mock/HLF, 520d/HLF [3 days after transfection (3D) and 5 days after transfection (5D)], 520d/HLF [7 days after transfection (7D)], 520d/HLF (R1 and R2), sorted with both GFP and alkaline phosphatase (ALP), iPSC (253G1), 520d-transfected and 253G1-derived progenitor hMSC (520d/hMSC), DNA extraction was performed using Qiagen DNeasy kit according to manufactures instruction (QIAGEN, Tokyo, Japan). Exome Sequencing with a Next-Generation Sequencer. Three replicates per group were analyzed (*n* = 3). Genomic DNA was processed using the SureSelectXT Human All Exon v5 + UTRs (Agilent Technologies, Inc.), and sequenced on the Illumina HiSeq 2500 platform with 101 bp paired-end reads (HSS, Sapporo, Japan). The paired-end reads in FastQ format were mapped to reference genome HG19 using BWA-0.7.10. The mapping files in SAM (Sequence Alignment/Map) format were converted to BAM (binary version of SAM) format and sorted by SAMtools-1.2. Local realignment around known indels was performed by GATK-Lite-2.3.0 on the sorted BAM files. Picardtools-1.133 [22, 23] was used to remove PCR duplicates. Finally, base quality score recalibration was performed using GATK again. RNA Sequencing with a Next-Generation Sequencer. Total RNA was processed using the TruSeq Stranded mRNA Library Prep Kit (Illumina), and sequenced on the Illumina HiSeq 2500 platform with 101 bp paired-end reads. The paired-end reads in FastQ format were mapped to reference genome HG19 using TopHat.

### Mutation analysis

Samtools mpileup [options: -d 10000 -L 10000 -B -t DP, DV, SP, DP4, DPR] was piped with bcftools call [options: -A -v -m -f GQ] to produce variant call format (VCF) files. VCF files were further filtered to select variants that had at least two reads supporting the variants and that showed a minimum allele frequency of 0.8 and a maximum read depth of 35 at called sites. For high-throughput, deep sequencing of selected cDNA such as *Tp53* were subjected to Sanger sequencing to screen for nonsynonymous mutations [24–26].

### RT^2^ PCR array analysis

To examine epigenetics-related genes that 520d-5p may regulate, PCR array analysis was performed using RT^2^ profiler PCR array (real-time PCR technology), according to manufacturers’ instruction (Qiagen Japan, Tokyo, Japan).

## Results

**Fig. 1:**
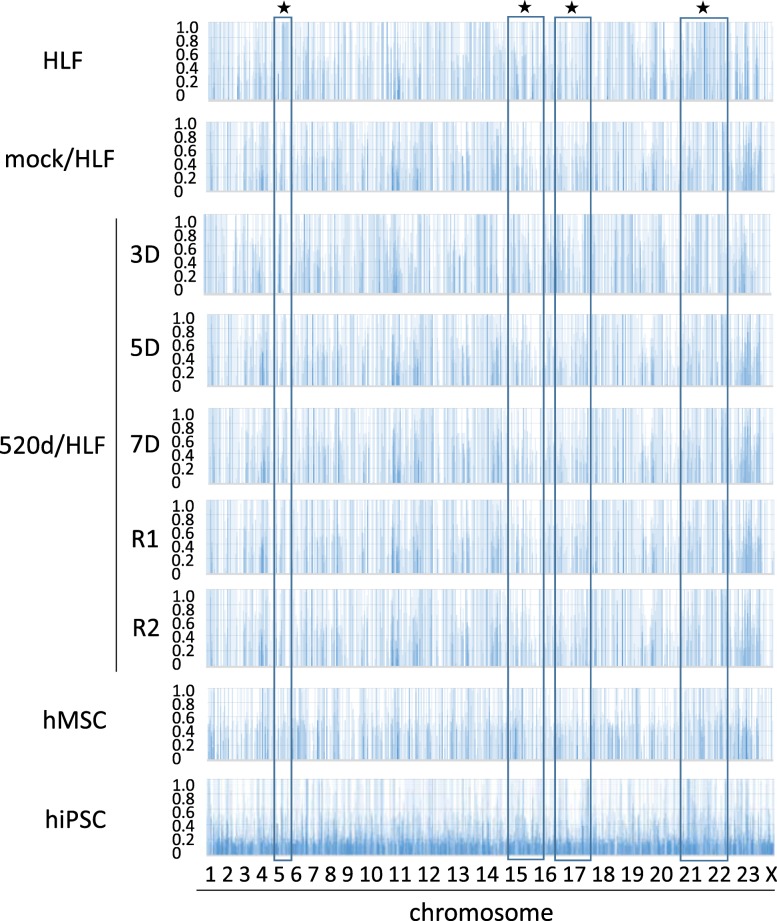
A frequency of allele changes per chromosome is shown. Mutation analysis in HLF, mock/HLF, 3D, 5D, 7D, R1, R2, hMSC, hiPSC by NGS. This area graph showed that the total number of mutations were getting reduced in HLF derivatives regarding a frequency of allele changes per chromosome. ★ indicates the representative sites in Fig. 3

**Fig. 2:**
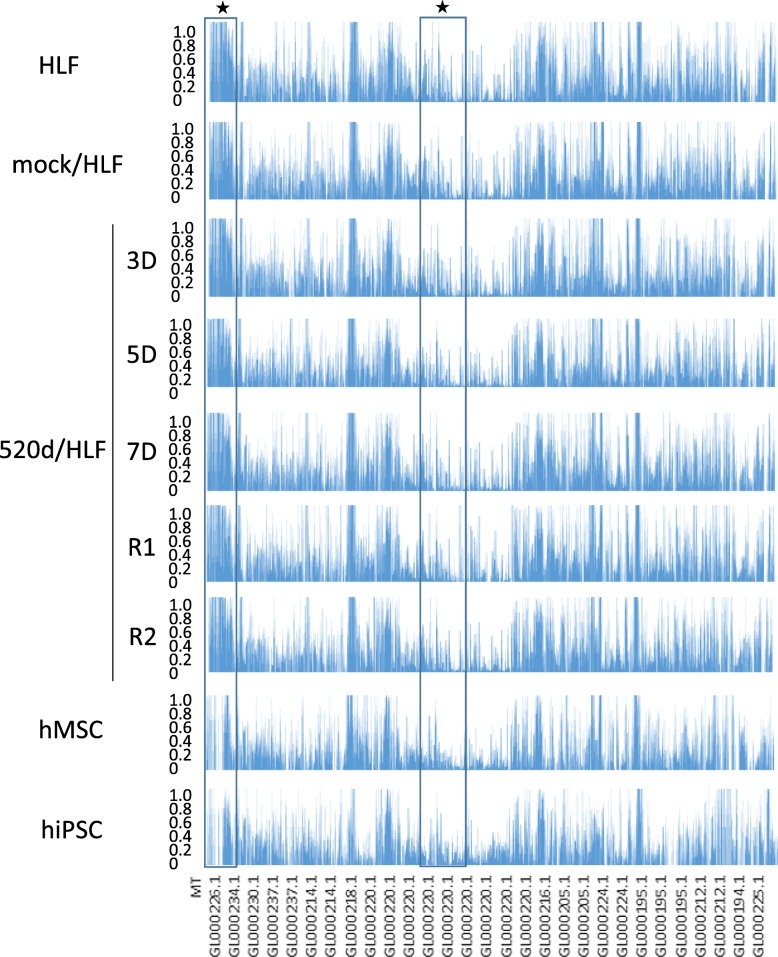
A frequency of allele changes of fragmented genomic sequence in which information was not decided on chromosome. Mutation analysis in HLF, mock/HLF, 3D, 5D, 7D, R1, R2, hMSC, hiPSC regarding a frequency of allele changes of fragmented genomic sequence by NGS. This area graph showed that the total number of mutations in genomic fragment assembly was reduced prominently. ★ indicates the representative sites in Fig. 3

**Fig. 3:**
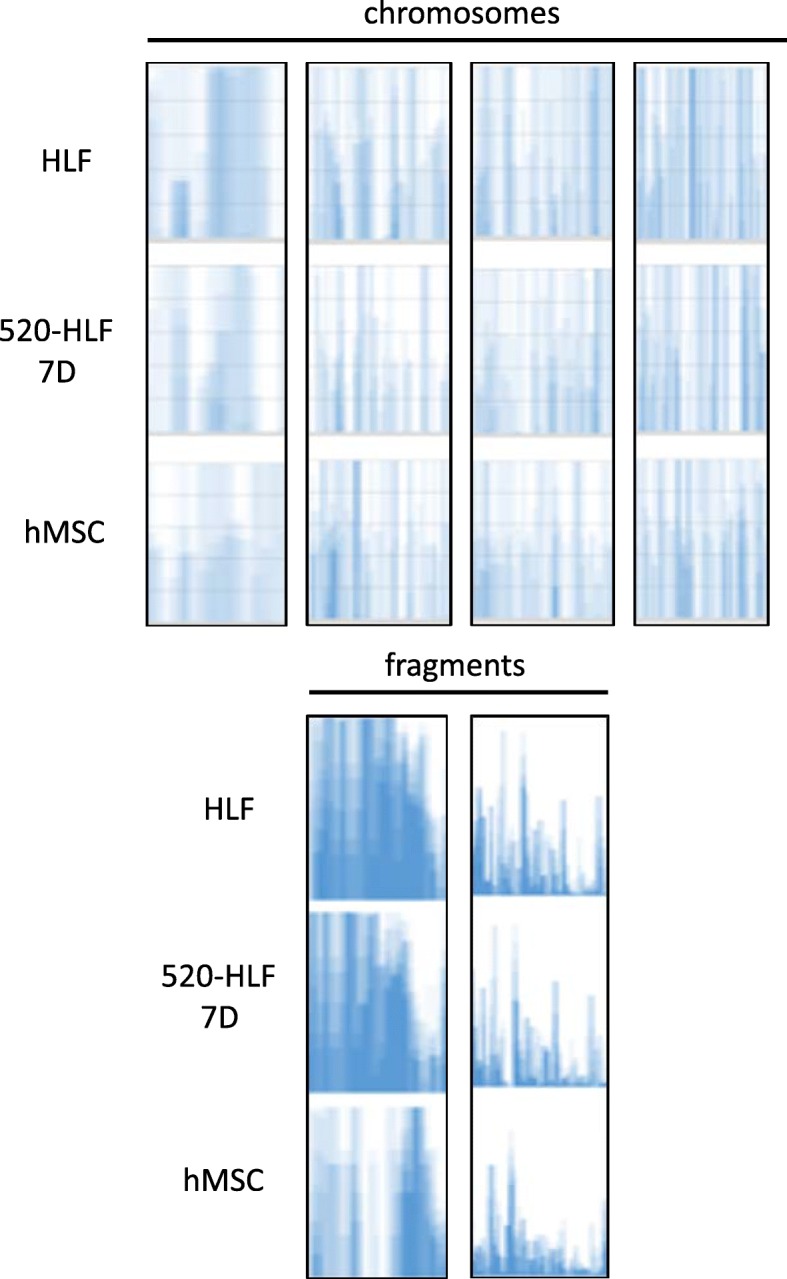
To show the genomic changes, 4 sites (upper) in Figs. 1 and 2 sites (bottom) in Fig. 2 were chosen and zoomed up in representative cells (HLF, 520d-5p 7D, and hMSC). The changes in genomic fragments were less than those of chromosomes. The chosen sites are shown as ★ in Figs. 1 and 2

Mutation analysis in HLF, mock/HLF, 3 days after transfection (3D), 5 days after transfection (5D), 7 days after transfection (7D), R1 (see ref. [17]), R2 (see ref. [17]), hMSC, 520d/hMSC or hiPSC showed that 520d-5p gradually decreased the total number of mutations, including point mutations, two-nucleotide mutations and three-nucleotide mutations (Figs. 1, 2). Mutations in genomic fragment assemblies were reduced more prominently than mutations in chromosomes assemblies (Figs. 1, 2 and Table 1). Four sites in chromosomes are shown in Fig. 1, and two sites in fragmented genomes are shown in Figs. 2 and 3. Furthermore, when we examined the frequency of genomic alterations in each chromosome, the reduction in mutation frequency was most prominently observed in chromosome X (Table 2). Interestingly, the increased number of frequencies seemed to be maintained with the reduction in number, except for in chromosome X.

**Table 1:** Details for genomic fragments that information was not decided on chromosome and shown at the bottom of the area graph in Fig. 2

MT (1–65)	
GL00026 (66–90)	
GL000229 (91–108)	
GL000231 (109–126)	
GL000239 (127–129)	
GL000201 (130–131)	
GL000246 (132)	
GL000249 (133)	
GL000238 (134–138)	
GL000234 (139–160)	
GL000232 (161–163)	
GL000240 (164–165)	
GL000241 (166–180)	
GL000243 (181–183)	
GL000230 (184–250)	
GL000237 (251–385)	
GL000204 (386)	
GL000198 (387–394)	
GL000208 (395–402)	
GL000191 (403–414)	
GL000228 (415–420)	
GL000214 (421–566)	
GL000221 (567–573)	
GL000209 (574–643)	
GL000218 (644–722)	
GL000220 (723–1345)	
GL000213 (1346–1347)	
GL000211 (1348)	
GL000199 (1349–1356)	
GL000217 (1357–1365)	
GL000216 (1366–1395)	
GL000205 (1396–1547)	
GL000219 (1548–1614)	
GL000224 (1615–1729)	
GL000223 (1730–1746)	
GL000195 (1747–1966)	
GL000212 (1967–2107)	
GL000222 (2108–2124)	
GL000193 (2125–2146)	
GL000194 (2147–2186)	
GL000225 (2187–2267)	
GL000192 (2268)

**Table 2:** The change (%) of frequency number in genomic alterations on each chromosome by 520d-5p between mock/HLF and 7D-transfectants

Status of frequency	chr. 1	chr. 2	chr. 3	chr. 4	chr. 5	chr. 6	chr. 7	chr. 8
reduction	8.5	9.4	10.8	7.2	5.4	14.5	3.7	10.4
no changes	83.2	81.3	78.8	85.6	89.4	71.4	92.7	79.6
gain	8.3	9.3	10.4	7.2	5.2	14.1	3.6	10.0
Status of frequency	chr. 9	chr. 10	chr. 11	chr. 12	chr. 13	chr. 14	chr. 15	chr. 16
reduction	3.9	11.4	9.7	11.0	3.1	4.8	4.4	12.5
no changes	92.3	76.9	80.5	77.9	93.0	90.5	91.4	74.7
gain	3.8	11.7	9.8	11.1	3.9	4.7	4.2	12.8
Status of frequency	chr. 17	chr. 18	chr. 19	chr. 20	chr. 21	chr. 22	chr. 23	chr. X
reduction	9.3	11.0	4.4	11.6	20.3	12.5	3.8	30.7
no changes	82.1	79.0	91.7	77.0	59.7	75.2	92.6	43.8
gain	8.6	10.0	3.9	11.4	20.0	12.3	3.6	25.5

**Fig. 4:**
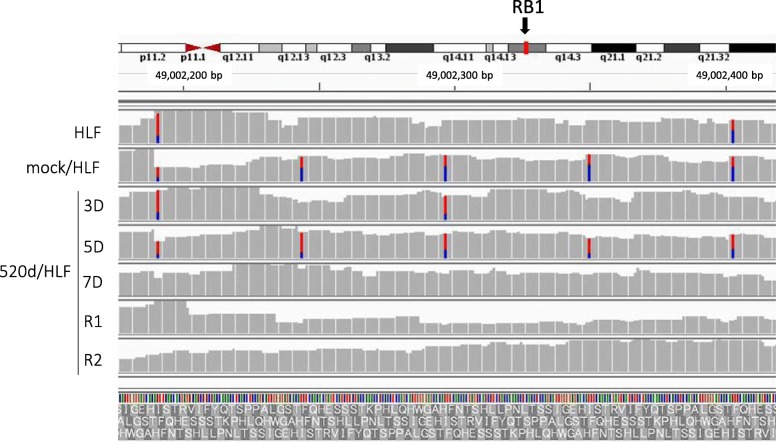
Representative conversion of RB1 after 520d-5p transfection. HLF originally had possible mutated regions at two sites, and alterations in 5 sites could be seen after transfection by 520d-5p. However, transfectants in 7D did not have any of them there. Conversion could be observed and maintained in R1 and R2 (sorted transfectants)

**Fig. 5:**
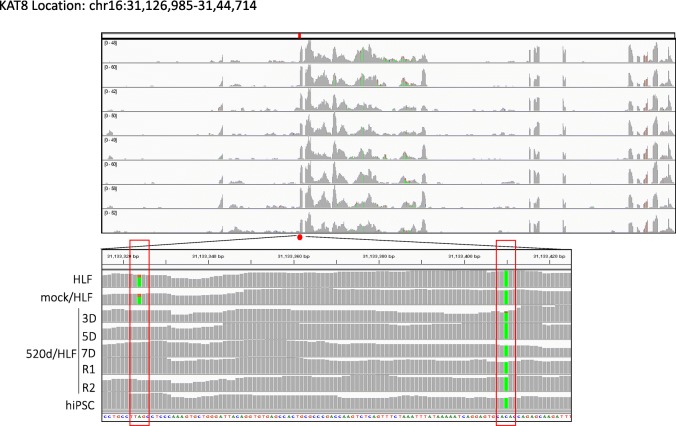
Representative conversion in *Kat8* after 520d-5p transfection. *Kat8* had two point mutations in HLF in RNA-seq, but this region had a conversion to wild type nucleotide since 3D after transfection by 520d-5p (left rectangle). All samples had the same and unchangeable nucleotide status in HLF to hiPSC (right rectangle). This figure was drawn based on the alignment data of RNA-Seq analysis

**Fig. 6:**
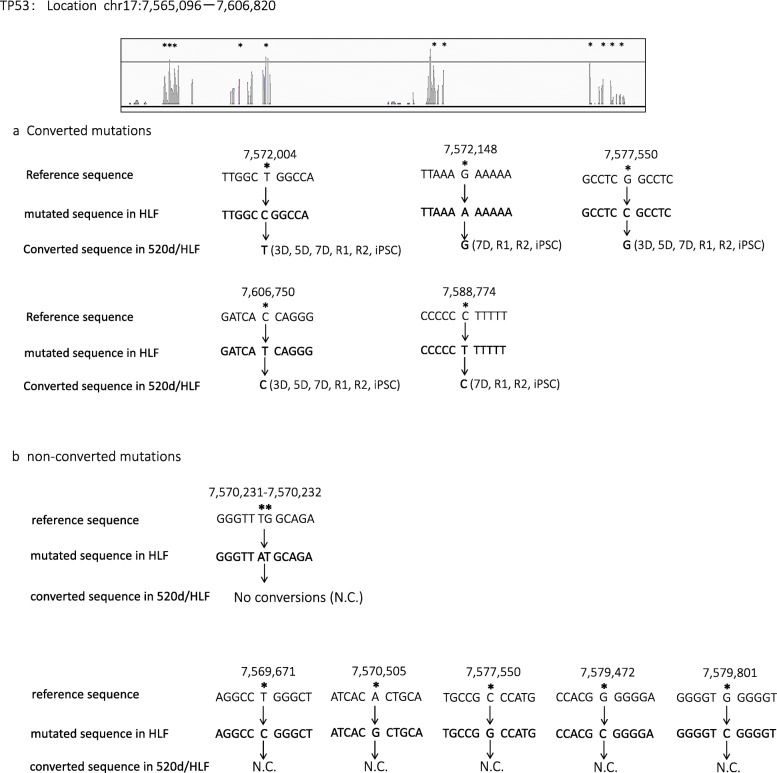
Analysis regarding the effect of 520d-5p on *Tp53* in mutated sites. Eleven mutated regions were found. **a** Five converted mutations to wild type status although the definite rule of conversion was not confirmed. **b** Remaining sites had no effects on mutation status in HLF and transfectants (N.C.). This figure was drawn based on the alignment data of RNA-Seq analysis. *means a mutated region

Conversions to wild-type nucleotides were observed in many genes. For example, NGS identified that the *Rb1* gene had four exon mutations. In two of the mutations, 520d-5p transfection caused base switching by 5D, and a base in the original site was wild type at 7D. The left and right bases were converted from T to C and C to T, respectively. The base conversion effect on three sites from 49,002,200 bp to 49,002,400 bp by lentiviral induction was reversible, and the mutations resolved after 7D (Fig. 4). Also, lysine acetyltransferase 1 (*Kat8*) had two mutations in HLF, but nucleotide mutations at two locations in *Kat8* (location: chr16:31,126,985-31,44,714) were reverted to wild type nucleotides by 520d-5p (Fig. 5). The nucleotide at location (31,188,324 in reference) in *Kat8* converted thymine in HLF and mock/HLF to adenine in 3D, 5D, 7D, R1, R2, and hiPSC. Also, *Tp53* had eleven mutations in HLF, and five of eleven point mutations were converted to wild type (Fig. 6a), while six mutation sites were not reverted to wild type (no conversions: N.C.) (Fig. 6b). The cell cycle regulator *Rb1* had four mutations in HLF and two of four mutations were converted to wild type (Fig. 4). Representative conversions of mutations in *Kat8/Myst1* (91,134,500 bp-91,135,000 bp on chr16 p11.2) are shown in Additional file 1: Figure S1. Allelic changes in representative genes related to DNA methylation, histone modification, DNA repair, oncogenesis, tumor suppression, stemness, and chromatin remodeling, and known target genes of 520d-5p, are shown in Additional file 2: Tables S1-S7.

**Fig. 7:**
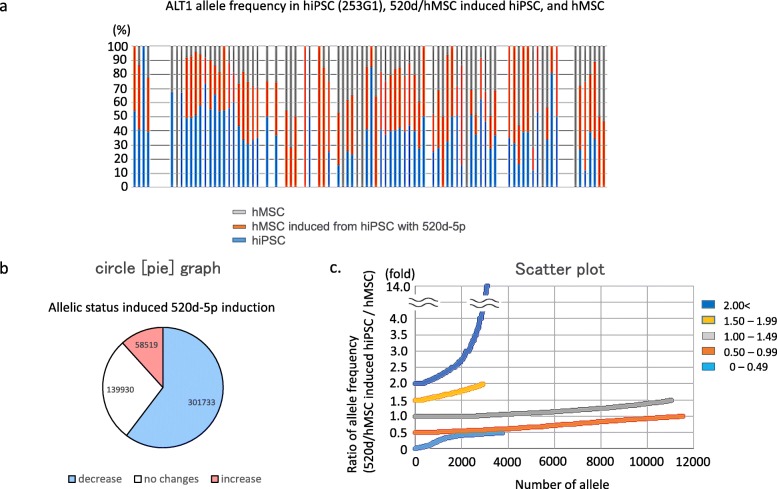
**a** ALT1 allele frequency in hiPSC, 520d/progenitor hMSC, or hMSC. hiPSC had non-negligible number of mutated sites, but the total number of mutations were reduced and some of them were converted to wild type during the differentiation process after 520d-5p transfection. **b** Circle graph showing allelic status induced by 520d-5p transfection. The decrease of ALT1 allele frequency was observed in 60.3% (301,733/500,189) of mutated sites. 11.1% (58,519/500,189) gained the new mutated regions. c. Scatter plot showing alterations of nucleotides during the differentiation towards hMSC status in the increased mutations (585,199 sites in 2)). Ratio of allele frequency 520d-5p-transfected hMSC progenitor cells / normal hMSC) was estimated. A large parts of alteration of increased sites in progenitor cells seemed to be an alteration accompanied by the differentiation process towards hMSC status according to the standardization to nucleotides status observed in hMSC

We were unable to detect a common mechanistic rule for conversion of mutations to wild type nucleotides. Mutations in 520d/hMSC derived from hiPSCs were clearly less than those of hMSCs (Fig. 7a), independent of individual differences. Approximately 60 % (60.3%; 301,733 out of 500,182) of nucleotide changes (allele frequency) in hiPSCs were reduced in hMSCs (520d/hMSC) both induced from hiPSCs and transfected by 520d-5p. 11.7% of mutations (58,519 out of 301,733) increased in 520d/hMSC (Fig. 7b). However, 38.6% of mutations (22,561/58519) of the change of allelic frequency in 520d/hMSC appeared to be nucleotide changes secondary to differentiation to hMSCs, as the 0.5 to 1.5 group indicated similar to allele frequency in hMSCs in volatility (Fig. 7c). The ratio of allele frequency was calculated by (520d/hMSC induced from hiPSCs/hMSC). Allelic changes in representative genes related to stemness are shown in Additional file 2: Table S7. *Kras*, *Cmyc*, or *Bcl2* in iPSC-derivatives had no changes in individual genes. *P21*, *Tp53*, and *Braf* had diminished resolution of mutated regions (Additional file 1: Figure S2-a, c, d). *Tp53* and *Stat3* (a target gene of 520d-5p) had alterations accompanied with differentiation (Additional file 1: Figure S2b). Additionally, mutations in two genes involved in DNA repair (*Abl2* and *Atr*) were decreased by 520d-5p transfection (Additional file 1: Figure S3).

PCR array analysis of epigenetics-related genes in 520d/HLFs (5D, 7D) and hiPSCs revealed that only two genes (*Hat1/Kat1* and *Myst1/Kat8*) were commonly downregulated by 520d-5p transfection (Additional file 1: Figure S4).

## Discussion

Previously, we reported that 520d-5p induces transformation of undifferentiated cancer cells into non-malignant status [17]. We have subsequently sought to determine the mechanism for this transformation. On the other hand, earlier studies concluded that cancer cell mutations do not revert to wild type status, and that cancer cell mutations are unaltered by anti-cancer agents or other factors, although one prior study suggested that BRCA mutations in triple-negative breast cancer correlate with miRNA expression [27, 28]. This study is the first to comprehensively demonstrate that genomic alterations or mutations reversibly changed to wild type. Here we demonstrated 520d-5p modulated cancer cell transformation from a malignant to benign phenotype in vivo using immunodeficient mice. This transformation was induced in the presence of genomic level mutations including exons, introns, and non-coding lesions.

Prior reports demonstrated that 520d-5p directly targets the 3’UTRs of eleven genes, including *Elavl2, Tead1, Gatad2b, Atm, Casp3, Tada3, Stat3, Twist1, Sirpa, Sp1* and *Cthrc1*, and more than 9000 genes are predicted targets of 520d-5p [17, 19, 20, 29–31].

**Fig. 8:**
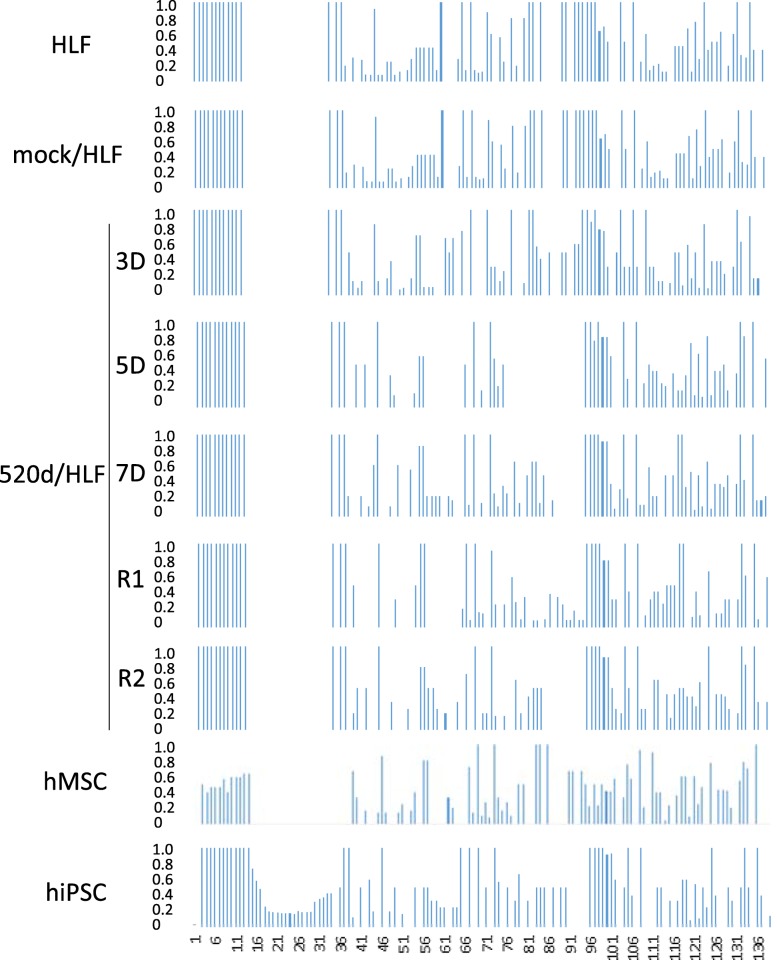
The improvement to wild type of ALT1 in HLF by the transfection of miR-520d-5p A bar graph showed that 520d-5p induced some of the ALT1 in transfectants, to wild type status

Together, current and previous finding demonstrate that 520d-5p has comprehensive effects on DNA and RNA demethylation, indicating that synergistic epigenetic alterations may be implicated in the cellular conversion phenomena via this reprogramming process. In the present study, we found that mutations greater than two-base alterations were not observed in undifferentiated hepatoma cells or iPSC derivatives expressing 520d-5p, suggesting that the effect of this miRNA is limited to one-base conversions, and appears to most prominently affect chromosome X (Fig. 8). However, we were unable to detect any specific rule for base conversion, for example T in reference to C in the samples, or C to T. Mutations and conversion status in genes related to DNA methylation, histone modification, DNA repair, oncogenesis, stemness, as well as chromatin remodeling and 520d-5p target genes and tumor suppressor genes, are summarized in Additional file 2: Tables S1-S7. However, the mechanism for these conversions remains unclear. We presume that the in-depth mechanism includes a synergistic process by RNA demethylation and methyltransferase accompanied with mutation reductions, independent of nucleotide type, as we suggested previously (17–20).

In comparison between hiPSCs and 520d-5p-transfected hMSCs induced from hiPSCs, we are interested in the effect of 520d-5p on hiPSCs that are not completely initialized. We attempted to differentiate hiPSCs towards hMSCs with four transfections of 520d-5p. As the result, we found that mutations in hiPSCs derived from original cells could be altered (Fig. 7a). These mutations had a dominant reduction (Fig. 7a, b), although new allelic alterations involved in the differentiation from hiPSC to hMSC increased (Fig. 7c). Because we identified that 520d-5p induced demethylation in HLF in previous studies [17, 19], we comprehensively examined epigenetics-related genes using a PCR array and identified indirect downregulation of *Hat1* and *Kat8,* which are lysine acetyltransferases [32–35]. However, the downstream functions of 520d-5p remain incompletely understood. To gain further insights and validate that our results are reliable in future studies, we will use additional approaches such as capillary sequencing, as there are fewer reading sites in the mutated alignments of other genes.

## Conclusions

These findings suggest that in cancer cells, malignant properties can be reprogrammed and reversed by induction of nucleotide conversion. These findings also suggest that 520d-5p has potential in regenerative medicine, as transfection improved the quality of hiPSCs.

## Additional files

Additional file 1: **Figure S1.** Representative conversion in *Kat8* after 520d-5p transfection. **Figure S2.** Comparative NGS analysis between hiPSC, 520d/hMSC progenitor cells, or hMSC. **Figure S3.** Comparative NGS analysis between hiPSC, 520d/hMSC progenitor cells, or hMSC regarding representative DNA repair genes. **Figure S4.** PCR array using RT2 Profiler PCR array system regarding epigenetics-related genes in 520d/HLF (5D, 7D) and hiPSC. (PDF 835 kb)

Additional file 2: Summary of nucleotides alterations in respective genes regarding of our interest (**Tables S1–S7**). (DOCX 26 kb)

## Data Availability

The datasets generated for this study are available from the corresponding author on reasonable request.

## Abbreviations

520d-5p: Hsa-miR-520d-5p
hiPSC: Human induced pluripotent stem cell
hMSC: Human mesenchymal stem cell
miRNA: MicroRNA
NGS: Next generation sequencing
