# Supplementary material for: miR-520d-5p can reduce the mutations in hepatoma cancer cells and iPSCs-derivatives
Source: BMC Cancer. 2019 Jun 15;19:587. doi: 10.1186/s12885-019-5786-y (PMC6570841; doi:10.1186/s12885-019-5786-y)

Supplementary Fig. 1

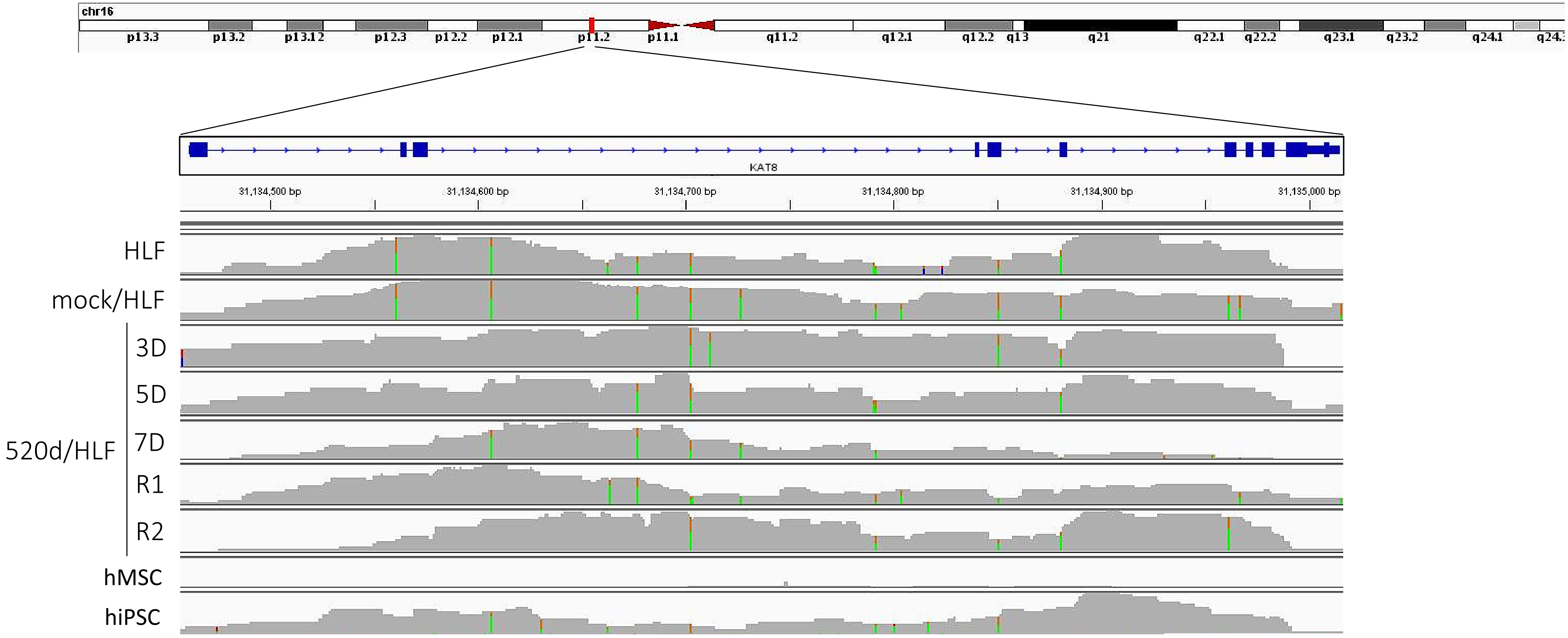

Supplementary Fig. 2

a. P21: reduction derived from 520d-5p transfection

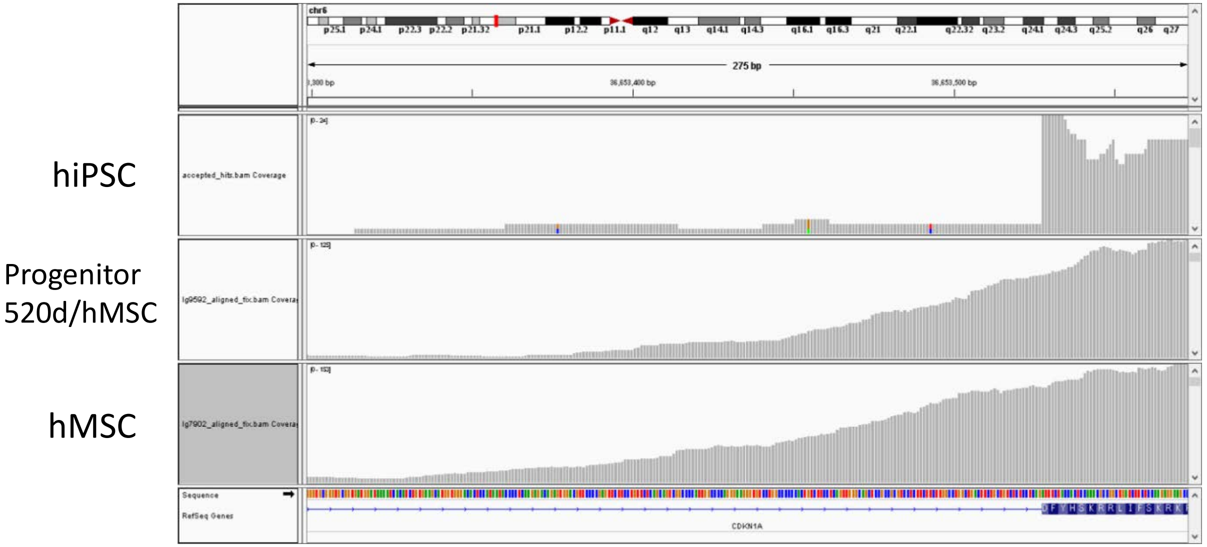

b. TP53 : alterations accompanied with the differentiation

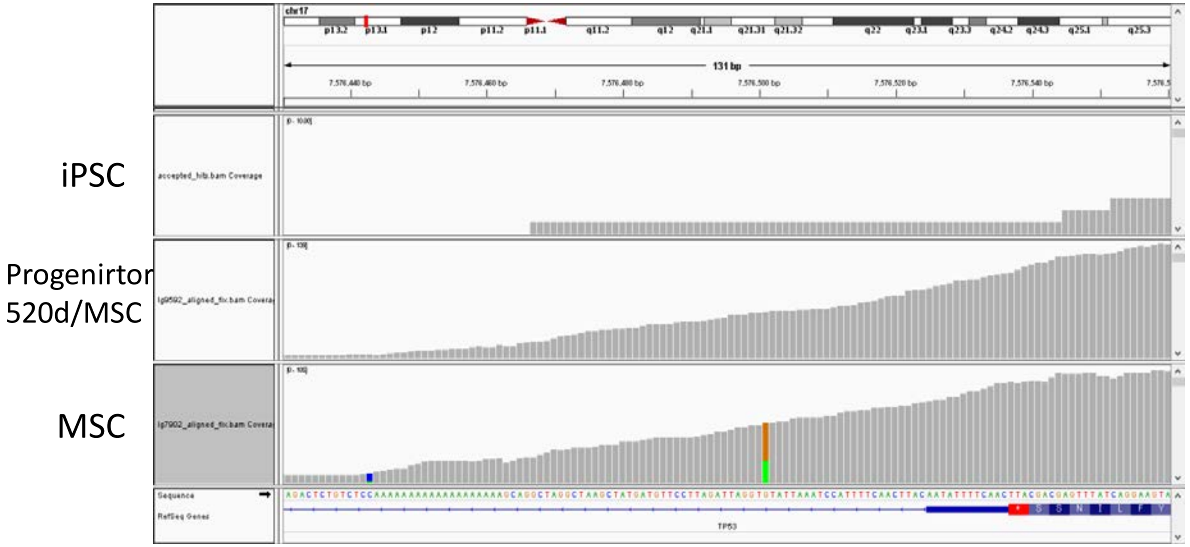

c. TP53: disappearance derived from 520d-5p transfection

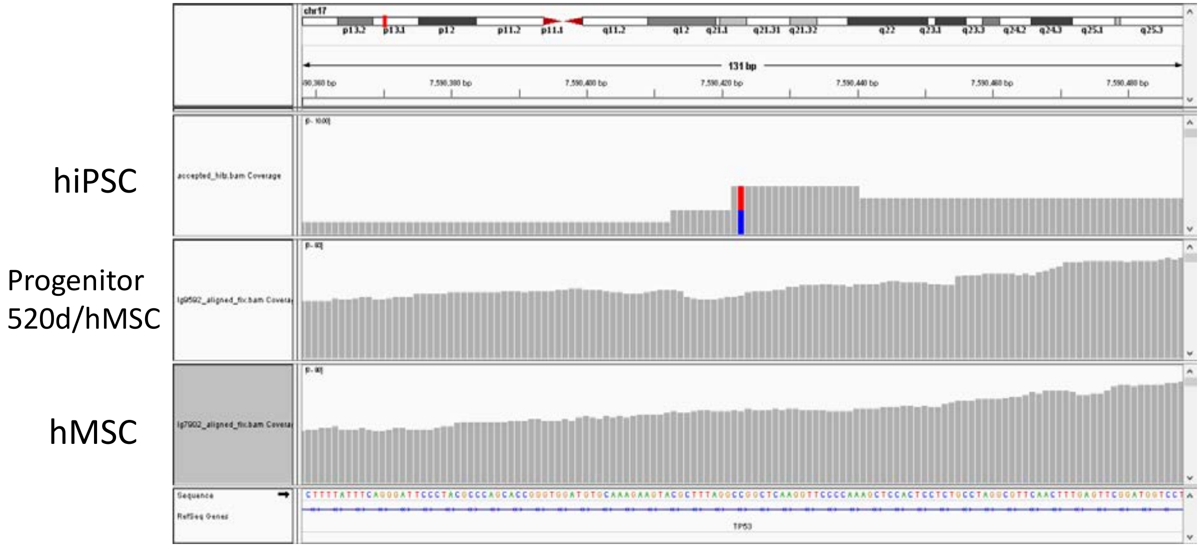

d. BRAF : reduction derived from 520d-5p transfection

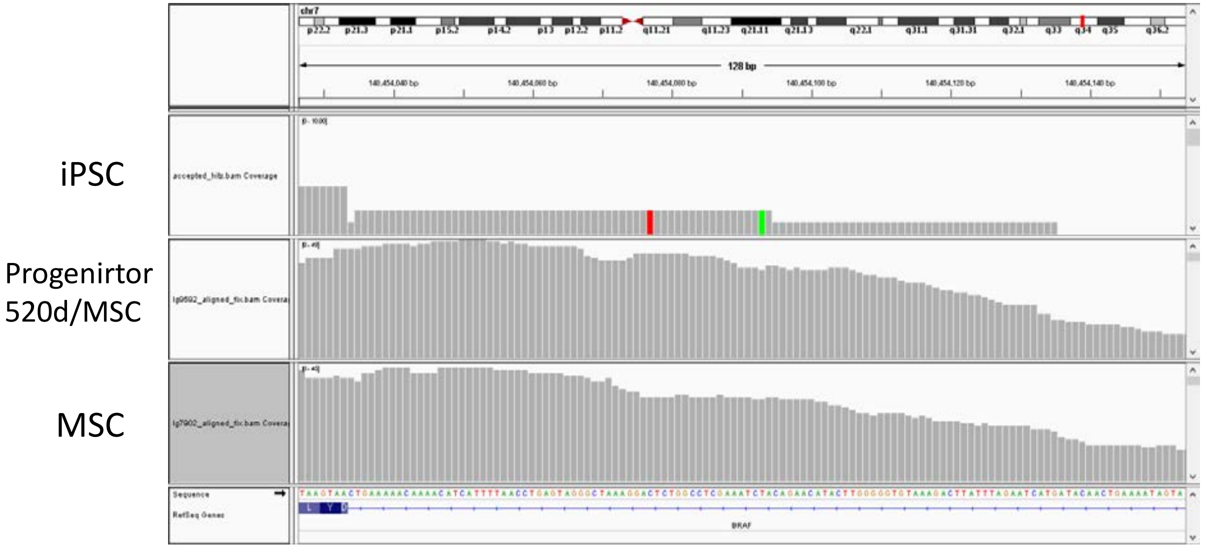

Supplementary Fig. 3

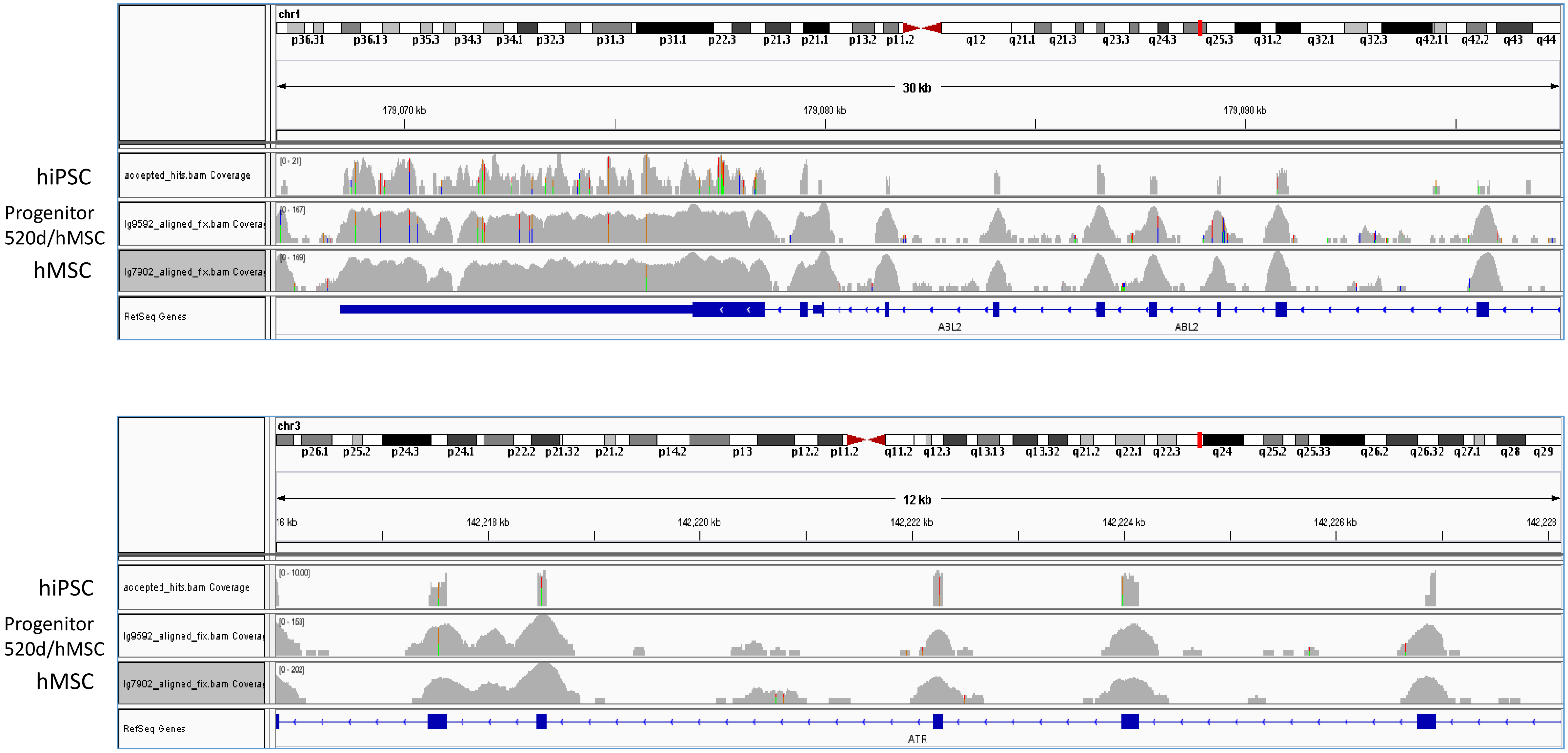

Supplementary Fig. 4

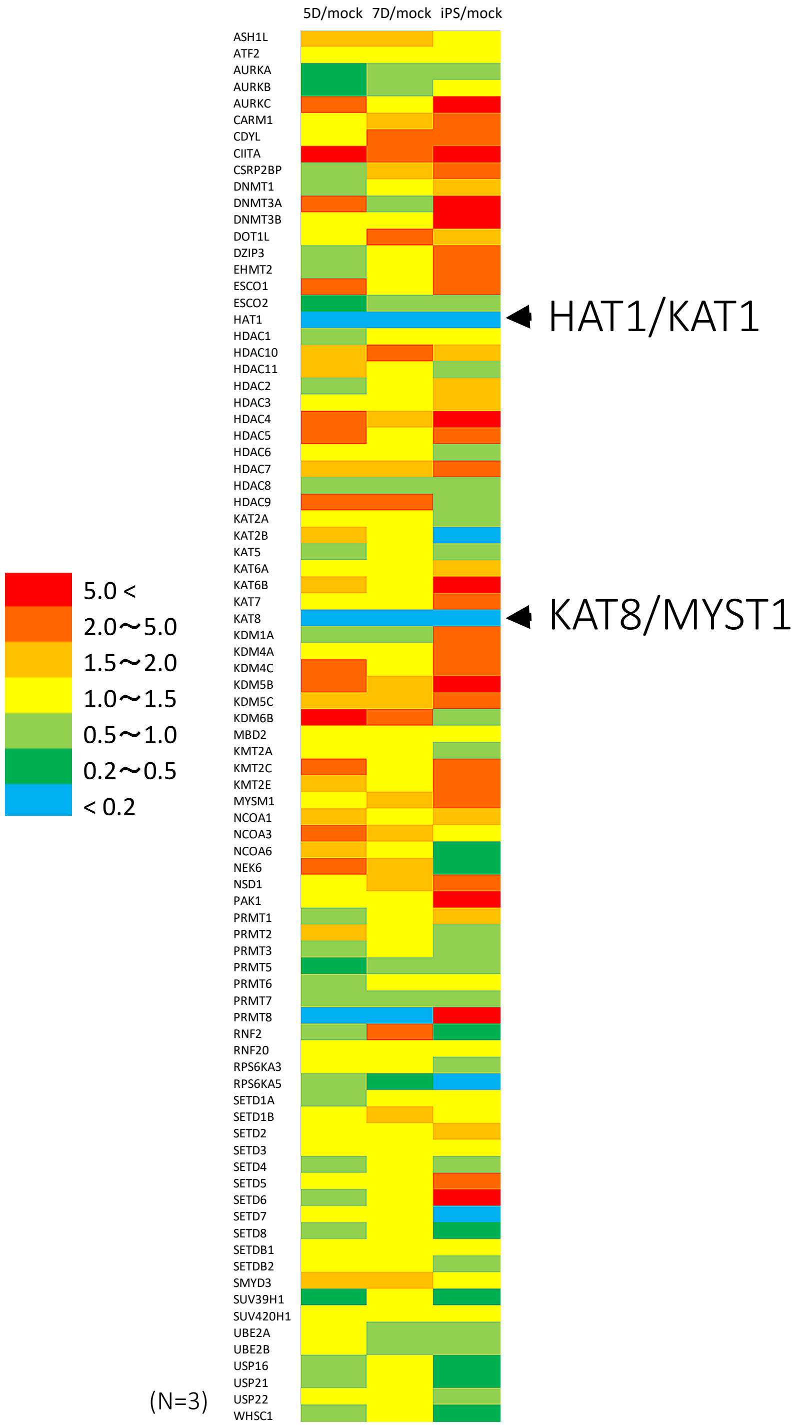

Supplement: Supplementary file 1 — Figure S1. Representative conversion in Kat8 after 520d-5p transfection. Figure S2. Comparative NGS analysis between hiPSC, 520d/hMSC progenitor cells, or hMSC. Figure S3. Comparative NGS analysis between hiPSC, 520d/hMSC progenitor cells, or hMSC regarding representative DNA repair genes. Figure S4. PCR array using RT2 Profiler PCR array system regarding epigenetics-related genes in 520d/HLF (5D, 7D) and hiPSC. (PDF 835 kb) [file 12885_2019_5786_MOESM1_ESM.pdf]
