# Supplementary material for: miR-520d-5p can reduce the mutations in hepatoma cancer cells and iPSCs-derivatives
Source: BMC Cancer. 2019 Jun 15;19:587. doi: 10.1186/s12885-019-5786-y (PMC6570841; doi:10.1186/s12885-019-5786-y)
Supplement: Supplementary file 2 — Summary of nucleotides alterations in respective genes regarding of our interest (Tables S1–S7). (DOCX 26 kb) [file 12885_2019_5786_MOESM2_ESM.docx]

Supplementary Table 1

DNA demethylation

| gene | Mutation in HLF | Mutation in iPSC | Mutation in MSC | Nucleotide change | Conversion by 520d-5p |
| --- | --- | --- | --- | --- | --- |
| AID | none | none | none | - | none |
| DNMT1 | 3 sites in exons | 3 sites in exons | 3 sites in exons | C to T, T to C | none |
| DNMT3A | 2 sites in exons | 2 sites in exons | 2 sites in exons | **T to C** (HLF) | Reversible conversion (conversion in 3D and 5D ) |
| DNMT3B | 3 sites in exons | 2 sites in exons | 1 site in exon | T to C (HLF, iPSC), A to T (MSC) | none |
| IDH1 | a site in exon | a site in exon | a site in exon | C to A | none |
| IDH2 | two sites in exons | none | 10 sites in intron | G to A & C to A (HLF) | none |
| MBD1 | a site in exon | a site in exon | none | C to A | none |
| MBD2 | none | a site in exon | a site in exon | C to A (MSC), A to C (iPSC) | none |
| TET1 | 10 sites in exons | 2 sites in exons | 3 sites in exons | C to T (iPSC, MSC), A to G (iPSC), T to A, | none |
| TET2 | none | none | none | - | none |

Supplementary Table 2

Histone modification

| gene | Mutation in HLF | Mutation in iPSC | Mutation in MSC | Nucleotide change | Conversion by 520d-5p |
| --- | --- | --- | --- | --- | --- |
| ASXL1 | 2 sites in exons | a site in exon | a site in exon | T to C (HLF) | possible conversion |
| BMI-1 | a site in exon | none | a site in exon | G to A | none |
| BRD4 | a site in exon | 2 site in exons | a site in exon | G to A, T to G (iPSC) | none |
| CREBBP | a site in exon | 2 sites in exons | a site in exon | C to T, T to C (iPSC) | none |
| EP300 | a site in exon | a site in exon | a site in exon | T to A | none |
| EZH2 | none | none | none | none | none |
| EHMT2/G9a | 3 sites in exons | a site in exon | 3 sites in exon | G to T, A to T, T to C (iPSC) | none |
| HAT1/KAT1 | none | none | none | none | none |
| HDAC2 | none | none | none | none | none |
| HDAC5 | a site in exon | a site in exon | a site in exon | G to C | none |
| HDAC7A | none | none | none | none | none |
| KDM1A/LSD1 | 7 sites in exons | 7 sites in exons | 7 sites in exons | T to G, T to C, C to T, G to A, G to T | none |
| KMT2A/MLL1 | 2 sites in exons | 3 sites in exons | 2 sites in exons | G to A, A to G (iPSC) | none |
| KMT2D/MLL2 | 5 sites in exons | a site in exon | 4 sites in exon | T to C, C to A, C to T (HLF), C to T (MSC), G to A (HLF) , G to A (HLF & MSC) | none |
| KMT2C/MLL3 | 9 sites in exons | a site in exon | 3 sites in exons | T to C, C to T, A to C, G to T, T to A, A to T, G to A (iPSC & HLF) | none |
| MYST1/KAT8 | 24 sites in exons | 10 sites in exons | 4 sites in exons | A to G , C to A, A to T, C to G (MSC), A to G (MSC), C to T (MSC) | possible conversion |
| PCAF/KAT2B | 4 sites in exons | a site in exon | a site in exon | C to A (MSC), T to C (iPSC) | none |
| PRMT1 | none | none | none | - | none |
| PRMT5 | none | none | none | - | none |
| SIRT1 | 4 sites in exons | 2 sites in exons | a site in exon | T to C, C to T(MSC & iPSC) | none |
| UTX/KDM6A | 6 sites in exons | 3 sites in exons | none | A to G (iPSC & 3D - R2), C to T, G to C, T to G, G to A (iPSC), C to G (iPSC) | none |

|  |
| --- |
|  |
|  |

Supplementary Table 3

DNA repair

|  | gene | Mutation in HLF | Mutation in iPSC | Mutation in MSC | Nucleotide change | Conversion by 520d-5p |
| --- | --- | --- | --- | --- | --- | --- |
|  | OTUB2 | 8 sites in exons | 5 sites in exons |  | T to C, G to A, C to T (iPSC), T to C, G to A, C to T, A to C, G to T (HLF), A to G, T to C (except MSC) | none |
|  | Abl2 | 8 sites in exons | 6 sites in exons | 4 sites in exons | A to G (all), T to G, G to T, G to A, C to T, A to G (except MSC), A to C (iPSC), C to T, A to G, A to T (HLF) , C to T, G to A, C to G (MSC) | none |
|  | ATR | a site in exon | a site in exon | a site in exon | C to T (all) | none |

Supplementary Table 4

Oncogenesis

|  | gene | Mutation in HLF | Mutation in iPSC | Mutation in MSC | Nucleotide change | Conversion by 520d-5p |
| --- | --- | --- | --- | --- | --- | --- |
|  | MYC | none | none | none | none | none |
|  | TERT | 2 sites in exons |  | none | C to A | none |
|  | TEP1 | 5 sites in exons | 4 sites in exons | 8 sites in exons | T to C (all), C to G, G to C, C to T (iPSC & MSC), C to T, G to A (MSC), A to G (except iPSC), C to A (HLF) | possible conversion |
|  | KRAS | 8 sites in exons | 7 sites in exons | 6 sites in exons | C to T, T to C, G to A (all), C to A, G to A (HLF), G to A (except R2, iPSC), T to C, A to G (except iPSC), A to C (iPSC, MSC) | possible conversion |
|  | HRAS | 4 sites in exons | a site in exon | a site in exon | A to G (all), A to G, C to T, T to G, G to A (HLF) | possible conversion |
|  | NRAS | none | none | none | none | none |
|  | BRAF | 2 sites in exons | 3 sites in exons | none | T to C, C to T, T to A (iPSC), T to G, T to C (HLF) | possible conversion |
|  | RAF1 | none | none | multiple sites in exons | | none |
|  | BCL2 | 5 sites in exons | a site in exon | 3 sites in exons | C to T, C to A (HLF), T to C, G to T (except iPSC), C to G (all) | Conversion |
|  | PI3K | none | none | none | -none | none |

Supplementary Table 5

Target gene

|  | gene | Mutation in HLF | Mutation in iPSC | Mutation in MSC | Nucleotide change | Conversion by 520d-5p |
| --- | --- | --- | --- | --- | --- | --- |
|  | TEAD1 | 5 sites in exons | 2 sites in exons | 5 sites in exons | C to G, T to A, G to A (HLF), A to G, C to T (all), A to G, C to T (MSC) | none |
|  | GATAD2B | 5 sites in exons | 4 sites in exons | 4 sites in exons | C to T, A to G, G to A (all), C to T (HLF) | none |
|  | ELAVL2 |  | 6 sites in exons | a site in exon | C to G (MSC), C to G, A to G, C to G, G to T, T to C (iPSC) |  |
|  | ATM | 6 sites in exons | 11 sites in exons, 4 sites in introns | 4 sites in exons | G to A (except MSC), A to G, A to T, T to C, A to T, G to T, G to A, C to T (iPSC), A to G, G to T (all), A to G, C to G, C to A, T to A (HLF) , C to T (MSC) | possible conversion |
|  | CASP3 | 5 sites in exons | 2 sites in exons | 5 sites in exons | G to T, A to G (except iPSC), T to C (all), A to T (MSC, iPSC), G to A , T to C (HLF), G to T (MSC) |  |
|  | TADA3 | none | 2 sites in exons | none | A to G, T to C (iPSC) | none |
|  | STAT3 | 8 sites in exons | 6 sites in exons | 5 sites in exons | T to C, C to T, G to A (HLF), G to A, A to C (iPSC) G to A, A to C, A to G, G to T, A to T (MSC) | possible conversion |
|  | TWIST1 | 5 sites in exons | 2 sites in exons | 3 sites in exons | C to G , A to G (iPSC), C to A (MSC) | none |
|  | SIRPA | 14 sites in exons | 8 sites in exons | 9 sites in exons | T to A, A to G, G to T (MSC, iPSC), C to T, A to G, G to A, T to C (HLF), G to A, G to C, C to G, C to T, T to C (all) | possible conversion |
|  | SP1 | 3 sites in exons | an exon in exon | a site in exon | C to T (MSC), C to T (iPSC), T to C (HLF) | possible conversion |
|  | CTHRC1 | 2 sites in exons | an exon in exon | a site in exon | C to A (MSC), T to C (all) | none |

Supplementary Table 6

Tumor suppressor gene

|  | gene | Mutation in HLF | Mutation in iPSC | Mutation in MSC | Nucleotide change | Conversion by 520d-5p |
| --- | --- | --- | --- | --- | --- | --- |
|  | RB1 | 4 sites in exons | none | a site in exon | A to G, T to C (HLF), C to T (MSC) | possible conversion |
|  | BRCA1 | 2 sites in exons | 8 sites in exons | 2 sites in exons | C to A, A to G, C to T, T to C, G to A (iPSC), A to T, A to G (HLF), T to C, G to A, A to G (MSC) | none |
|  | BRCA2 | 12 sites in exons | a site in exon | 3 sites in exons | T to C, T to A (all), A to T, T to C, A to G, A to C, G to A, T to G, C to A, C to T (HLF), C to A, G to A, T to G (MSC) | possible conversion |
|  | NF1 | 5 sites in exons | 2 sites in exons | 3 sites in exons | A to G (HLF), G to A, T to C (all), C to T, T to C, G to A (MSC) | none |
|  | NF2 | 4 sites in exons | 3 sites in exons | 3 sites in exon | G to C (all), A to G (iPSC), T to C, C to G (except iPSC), G to T (except MSC) | none |
|  | WT1 | none | none | none | none | none |
|  | VHL | 9 sites in exons | 8 sites in exons | 8 sites in exons | T to G, G to A, A to G, C to T, T to C (all), G to C (HLF) | conversion |
|  | APC | 6 sites in exons | 2 sites in exons | 3 sites in exons | C to A, T to C, T to G (HLF), A to T, C to G (iPSC), G to A, T to G, T to A (except iPSC) | possible conversion |
|  | CDKN2A/p16 | 5 sites in exons | 4 sites in exons | a site in exon | A to G (HLF), C to G (all), C to A, T to A, A to G (except MSC) | possible conversion |
|  | SERPINB5/Maspin | deletion | deletion | 10 sites in exons | T to G, C to A, G to A, G to C, A to C, A to T | - |
|  | TP73 | none | 5 sites in exons | 9 sites in exons | C to T, T to C, G to A, A to G, C to G (iPSC, MSC), A to G, G to A, C to T, T to C (MSC alone) | none |
|  | PTEN | a site in exon | a site in exon | a site in exon | G to A | none |
|  | SMAD4/DPC4 | none | none | none | - | none |
|  | CHEK2 | none | none | 2 sites in exons | A to T | none |
|  | MSH2 | none | none | none | - | none |
|  | MLH1 | none | none | none | - | none |
|  | PMS2 | 8 sites in exons | 4 sites in exons | 3 sites in exons | G to A, T to C, C to G, A to G, G to A, T to C (HLF), T to C, C to G, A to G (iPSC), T to C,(all), G to C, G to A (except iPSC) | possible conversion |
|  | DCC | deletion | 2 sites in exons | 6 sites in exons | T to C, A to G (iPSC), C to G, C to A, T to C, C to T, G to T, A to T (MSC) | - |
|  | SDHD | a site in exon | 2 sites in exons | a site in exon | T to C, A to G (iPSC), G to T (MSC), A to G (except iPSC) | none |
|  | CDKN1C/p57KIP2 | none | none | none | - | none |

Supplementary Table 7

Stemness and differentiation

| gene | Mutation  in iPSC | Mutation  in derivatives | Mutation  in MSC | Mutation in both derivatives and MSC | Nucleotide change by 520d-5p (bold: conversion) |
| --- | --- | --- | --- | --- | --- |
| NANOG | 7 sites | 8 sites | 6 sites | 6 sites | A to G, C to T, **C to A** |
| POU5F1 | 10 sites | 17 sites | 12 sites | 4 sites | G to T, **G to C**, T to C, C to A, **C to T** |
| KLF4 | 5 site | none | none | none | **G to A, T to C, G to A** |
| SOX2 | 1 site | none | none | none | **A to T** |
| MYC | 5 sites | 3 sites | 3 sites | none | C to G, C to A, **T to C, A to G** |
| TERT | 11 sites | 5 sites | 7 sites | 5 sites | A to T, A to C, **G to A, T to C** |
| ITGB1/CD29 | 15 sites | 18 sites | 11 sites | 8 sites | **T to C, C to A**, **C to T** |
| NT5E/CD73 | 7 sites | 10 sites | 4 sites | 3 sites | none |
| THY1/CD90 | 8 sites | 10 sites | 5 sites | 3 sites | G to T, G to C, **T to C**, C to T, **A to C**, A to G, T to G |
| ENG/CD105 | 22 sites | 28 sites | 17 sites | 17 sites | **T to A**, **G to C**, C to G, **A to G**, **C to T**, **C to A**, T to G |
| ALCAM/CD166 | 10 sites | 17 sites | 15 sites | 8 sites | T to G, T to A, **C to A**, A to T, **G to A**, **A to G, T to C** |

Bold: possible conversion by 520d-5p
